# United States Military Tropical Medicine: Extraordinary Legacy, Uncertain Future

**DOI:** 10.1371/journal.pntd.0002448

**Published:** 2013-12-26

**Authors:** Coreen M. Beaumier, Ana Maria Gomez-Rubio, Peter J. Hotez, Peter J. Weina

**Affiliations:** 1 Departments of Pediatrics and Molecular Virology and Microbiology, National School of Tropical Medicine, Baylor College of Medicine, Houston, Texas, United States of America; 2 Sabin Vaccine Institute and Texas Children's Hospital Center for Vaccine Development, Houston, Texas, United States of America; 3 James A. Baker III Institute for Public Policy, Rice University, Houston, Texas, United States of America; 4 Walter Reed Army Institute of Research, Silver Spring, Maryland, United States of America; Sanaria Inc., United States of America

*Throughout the 20^th^ century and into this new millennium, American troops in combat have been devastated by tropical infections. In response, the United States military has assembled an essential scientific and public health capability to combat these diseases. But the legacy of military tropical medicine now benefiting many aspects of global health is under threat.*

Over the last hundred years the morbidity suffered by US troops engaged in conflict as a result of tropical infections has in some cases exceeded combat casualties [1]. The specific tropical infections that occurred in each of the major US engagements are summarized in Table 1.

**Table 1:** Major tropical diseases in US military wars and conflicts.

War/Conflict	Years	Major Areas	Tropical Disease	Estimated or Reported Number of Cases	Ref.
World War I	1917–18	Americas and Caribbean	Malaria	27,203 malarial admissions	[1], [2]
World War II	1942–45	South Pacific, especially New Guinea, the Philippines, other Pacific Islands	Dysentery and diarrhea	756,849	[1]
			Malaria	572,950	[1]
			Dengue	121,608	[1]
			Hookworm	19,943	[1]
			Lymphatic filariasis	14,009	[1]
			Sandfly fever	12,634	[1]
			Scrub typhus	7,421	[1]
			Amebic dysentery	4,504	[1]
			Schistosomiasis	1,672	[1]
			Endemic typhus	893	[1]
			Leishmaniasis (*Leishmania* spp.)	361	[1]
			Strongyloidiasis	Not determined	[5]
Korean War	1950–53	Korea	Malaria	>34,864 malarial admissions	[2]
			Hantaan virus	1,600 cases of renal syndrome	[7]
			Japanese encephalitis	Not determined	[8]
Vietnam Conflict	1964–73	Vietnam	Malaria	65,053 malarial admissions	[2]
			Dengue	Up to 80% of fevers of unknown origin	[3]
			Japanese encephalitis	Not determined	[8], [9]
			Chikungunya		[6]
			Hepatitis A	Not determined	[10]
			Scrub typhus	Not Determined	[6], [9]
			Melioidosis	Not Determined	[6], [9]
			Leptospirosis	Not Determined	[6], [9]
			Amebic dysentery	Not Determined	[6]
			Hookworm and strongyloidiasis	Not Determined	[6], [9]
Operation Urgent Fury	1983	Grenada	Hookworm	>20%	[11]
Fort Sherman Jungle Training	1980s	Panama	Cutaneous leishmaniasis (*L. braziliensis panamensis*)	Not Determined	[12]
Operations Desert Shield and Desert Storm	1990–91	Iraq, Kuwait, Saudi Arabia	Diarrhea, predominantly ETEC and *Shigella sonnei*	>50%	[13]
			Cutaneous leishmaniasis (*L. major*)	19	[13], [14]
			Visceral leishmaniasis *(L. tropica)*	12	[13], [14]
			Malaria	7	[13], [14]
			Q fever	3	[14]
UN Operation Restore Hope	1992–93	Somalia	Malaria	112 (97 from *Plasmodium vivax*)	[17]
			Dengue	Not determined	[3]
Operation Uphold Democracy	1994	Haiti	Dengue	342 seropositive by IgM	[3]
Operations Enduring Freedom, Iraqi Freedom, New Dawn	2001–present	Afghanistan, Iraq	Diarrhea, predominantly ETEC and enteroaggregative *E. coli*	77% Iraq; 54% Afghanistan	[18], [19]
			Q fever	Outbreaks in Iraq	[18]
			Malaria (predominantly *P. vivax*)	Vivax malaria attack rate of 52.4 cases per 1,000 soldiers among Army Rangers deployed to eastern Afghanistan	[18], [19]
			Cutaneous leishmaniasis (*L. major*)	0.23% of deployed US ground forces in Operation Iraqi Freedom; 2.1% among a 2004 survey of 15,549 US military personnel deployed to one or more operations	[18]–[20]
			Cutaneous leishmaniasis (*L. tropica, L. infantum-donovani, L. tropica*)	Undetermined number of cases in Afghanistan; 2.1% among a 2004 survey of 15,549 US military personnel deployed to one or more operations	[18]–[20]
			Visceral leishmaniasis (*L. infantum-donovani*)	At least 9 cases	[18], [21]
			Brucellosis	3 cases	[18]
Operation Sheltering Sky and USAID efforts	2003, 2009	Liberia	Malaria (predominantly *Plasmodium falciparum*)	45 Cases	[22], [23]

## 1917–1945: World War I and World War II

Allied forces in the Middle East and East Africa suffered heavily from malaria and from diarrheal disease and dysentery in World War I [1]. Among American troops during 1917 and 1918, malaria accounted for approximately 27,000 hospital admissions [2]. Ironically, many of these infections were acquired among US naval forces in the Caribbean region and adjacent regions of the Americas, in addition to some of the malaria-endemic areas of the American South [2].

In World War II, hundreds of thousands of American troops serving in the Pacific theatre were struck by tropical infections, especially those returning from New Guinea and the Philippines [1]. In all, almost one million cases of tropical infections occurred among US troops [1]. Diarrheal disease and dysentery were widespread, and Mackie (1947) estimated that approximately one in four returning GIs suffered from at least one tropical infection, led by intestinal protozoa (mostly *Entamoeba histolytica*) or helminths (mostly hookworm infection), as well as relapsing malaria from *Plasmodium vivax* (and a significant number of *P. falciparum* infections) [1]. Schistosomiasis caused by *Schistosoma japonicum* was also common among soldiers fighting in Leyte, Philippines [1]. Many of these diseases were latent and were not diagnosed until American soldiers returned to the US.

Malaria was particularly widespread in the Pacific theatre—although transmission also occurred in southern Europe and North Africa—with more than 500,000 *Plasmodium* spp. recorded infections [1]. The impact of malaria was summarized by Beadle and Hoffman [2]. On Guadalcanal in the Solomon Islands, every man who served acquired malaria, and on average more than 5,000 soldiers were on the sick list daily because of malaria, especially the marines [2]. General Douglas McArthur once said to Dr. Paul F. Russell, Col US Army Chief of the Malaria Control Branch, “this will be a long war if for every division I have facing the enemy I must count on a second division in the hospital with malaria and a third division convalescing from this debilitating disease!” [2].

Two other vector-borne infections predominated in the Pacific theatre: dengue and lymphatic filariasis [3], [4]. Lymphatic filariasis forced the evacuation of large numbers of troops from New Guinea and the Tonga Islands, costing the US military an estimated $100 million [4]. A significant percentage of US prisoners of the Japanese in the Philippines and elsewhere also acquired tropical infections including malaria, strongyloidiasis, and nutritional deficiencies leading to neuropathies and cardiac beriberi [1], [5].

## 1950–1973: Korean and Vietnam Conflicts

More than 4,000 cases of *P. vivax* malaria struck US troops in the early part of the Korean conflict, especially during the defense of the Pusan perimeter, the Inchon landing, and the withdrawal from the Yalu River [2]. In contrast, *P. falciparum* malaria was the predominant form of malaria in the Vietnam conflict, with almost 25,000 cases and 50 deaths [2], [6]. The Marine Corps was considered especially vulnerable with a significant rise of cases between 1966 and 1969 as military tactics shifted to sorties into isolated rural areas [2]. Dengue was also a serious problem among US troops deployed to the rural Mekong River Delta and elsewhere, and accounted for a high percentage of fever of unknown origin [3]. The Korean war also led to 1,600 veterans falling ill with hemorrhagic fever with renal syndrome due to infection with Hantaan virus [7] in addition to significant numbers of infections with Japanese encephalitis virus [8]. Among the other tropical infections noted to occur among US soldiers in Vietnam and Vietnam veterans were Japanese encephalitis, hepatitis A, Chikungunya, scrub typhus, melioidosis, leptospirosis, amoebiasis, hookworm, and strongyloidiasis [6], [8]–[10].

## The 1980s: Operation Urgent Fury and Fort Sherman

During the Grenada invasion of 1983, more than 20% of US troops contracted hookworm infection as a result of being bivouacked on grounds contaminated with third-stage infective *Necator americanus* larvae [11]. Cutaneous leishmaniasis caused by *Leishmania braziliensis panamensis* was also a significant problem for US troops engaged in jungle training at Fort Sherman in Panama [12].

## 1990–91: Operations Desert Shield and Desert Storm

Immediately prior to the Iraqi invasion, US troops were transported to desert locations in northeastern Saudi Arabia and elsewhere in the Persian Gulf shortly after Iraq invaded Kuwait in 1990 [13]. More than 50% of troops fighting in Operations Desert Shield and Desert Storm reported an episode of diarrhea, with the leading etiologic agents being enterotoxigenic *Escherichia coli* (ETEC) and *Shigella sonnei* that were mostly acquired from ingesting locally grown produce [13], [14]. During the preceding Iran-Iraq war, cutaneous leishmaniasis (CL) was widespread [15], so it was anticipated that this condition would become an important problem among US troops. Unexpectedly, a dozen troops also acquired visceral leishmaniasis (VL) from *Leishmania tropica* infection [13], [14]. However, overall the number of sandfly-transmitted illnesses, including CL, VL, and sandfly fever, was lower than expected possibly because the peak period of troop buildup occurred during the cooler winter months of the year [13], [14]. This observation may explain why Rift Valley fever and Crimean-Congo Hemorrhagic fever were not significant tropical infections despite the presence of the arthropod vectors that transmit these viral infections [16].

## 1992–94: United Nations Operation Restore Hope and Operation Uphold Democracy

American troop involvement in Somalia (Operation Restore Hope) resulted in 112 cases of malaria (mostly *P. vivax*) among US troops [17]. This operation as well as the subsequent invasion of Haiti in 1994 (Operation Uphold Democracy) [3] also resulted in a significant dengue problem.

## 2001–Present: Operations Enduring Freedom, Iraqi Freedom, New Dawn, and Sheltering Sky

Overall, non-battle injuries were six times more common than battle injuries during the 21^st^ century conflicts in Iraq and Afghanistan [18]. As in the Persian Gulf conflicts in the 1990s, most personnel experienced at least one episode of diarrhea with ETEC and enteroaggregative *E. coli* as the leading etiologic agents [18]. *P. vivax* malaria was an important disease with an incidence of 52.4 cases per 1,000 soldiers. Malaria was diagnosed with a median time of 233 days after return to the US [18]. CL was also widespread, occurring in as many as 2% of troops deployed to Afghanistan and Iraq [19]. Troops deployed to Iraq acquired CL predominantly from *L. major* infection [18], while troops in Afghanistan were infected with both CL caused by *L. tropica* and *L. major*, and VL from *L. infantum-donovani* [18], [20]. Of particular concern were the effects of anti-leishmania therapy for *L. tropica* and *L. infantum-donovani* infections and whether reactivation of these infections will become widespread among veterans of the Afghanistan conflict [18], [20], [21]. While deployed to Liberia during Operation Sheltering Sky in 2003, cases of *P. falciparum* malaria occurred in 44 Marines (14 confirmed and 30 presumptive cases) out of the 225 total Marines deployed, resulting in a 20% attack rate which occurred within ten days of arrival in Liberia [22]. *P. falciparum* claimed the life of Naval Petty Officer Joshua Dae Ho Carrell, a Seabee deployed during USAID efforts in 2009 in Liberia [23].

## Summary Statement

The major tropical infections acquired by American troops during conflict over the last few decades include: 1) intestinal infections led by bacterial agents of diarrhea and dysentery, amebiasis and amebic dysentery, and two soil-transmitted helminthiases, i.e., hookworm infection and strongyloidiasis; and 2) key vector-borne infections including *P. falciparum* and *P. vivax* malaria, dengue and other arbovirus infections, and both forms of leishmaniasis—CL and VL. Consistently throughout the 20^th^ and into the 21^st^ century, the health and military impact of these tropical infections has approached or exceeded that resulting from battlefield injuries. Furthermore, in April 2010, the US Army Medical Research and Materiel Command hosted a panel of experts to determine the top priorities of infectious disease threats to the US military which highlighted many of those listed above (e.g., malaria, dengue, bacterial diarrheal diseases, and leishmaniasis) and further underscored the continued critical need for research and product development to combat these pathogens [24].

## America's Response and Future Directions

In response to the substantive burden of tropical infections during war and for years afterwards, the US military has consistently worked to develop new disease control tools, including drugs, diagnostics, and vaccines. Ultimately, the discoveries made by the major organizations committed to the US military's tropical medicine research and development (R&D) enterprise, including the Walter Reed Army Institute of Research (WRAIR), the Naval Medical Research Center (NMRC), and their affiliated overseas units, significantly affect our warfighters and protectors while simultaneously aiding and empowering the world's poor who are also plagued by these debilitating diseases. One of the most critical interventions developed by the US military was the development by Capt Robert Phillips of IV therapy to combat cholera and drastically decrease the fatality rate from 60% to <1% which earned him the Lasker Award in 1967. [25]. In addition, many life-saving interventions have already been licensed such as mefloquine, Malarone, and the hepatitis A and Japanese encephalitis vaccines. Other promising measures that are in clinical trials are RTS,S malaria vaccine, as well as new vaccines for adenovirus infection, dengue, and even HIV/AIDS. WRAIR, NMRC, and their affiliated overseas laboratories also interface and work closely with civilian populations throughout the world to conduct high-impact surveillance and disease detection studies in research areas such as multidrug-resistant bacteria (the Multidrug-Resistant Surveillance Network), leishmaniasis (the Leishmania Diagnostics Laboratory), and HIV diagnostics through a global network of sophisticated and accredited diagnostic laboratories.

Despite a legacy of US military tropical medicine and R&D and its vital impact on global health, its current efforts are in significant danger of being shut down. Due to deep congressional budget cuts, US military tropical medicine is under threat. At WRAIR alone in the last year, there have been more than one hundred contractors who lost work and two government worker reductions in force (RIFs) already due to the uncertainty and resulting lost revenue. The newest cuts have resulted in the inability to hire (a virtually complete hiring freeze and stringent government civilian ceilings) workers lost due to retirements while leaving the WRAIR workforce to find better and more secure work. More personnel reductions are certain with the “Sequester,” and existing programs are severely limited due to travel freezes and loss of confidence by key scientific and industrial partners. There are second, third, and fourth order effects of this downward spiral. Even the currently limited loss of personnel and travel have resulted in fewer public/private partnerships (with a resulting loss of matched funding), reduced cooperative R&D agreements, and decreased continued development of products and clinical trials as all “new starts” are frozen. The most devastating loss could be a pending decision to sacrifice much of the entire medical R&D effort (to “technology watch” status) in order to instead support immediate health care needs of returning warriors in the military medical system.

When the US military faced significant financial hardships and “peace dividends” in the past, they were able to continue their R&D missions through wise planning and measured reductions. They planted the seeds of recovery for the post-reduction period by making sure that there were still at least small investments in personnel and by working to maintain close relationships with private organizations that sustained R&D efforts through budget-lean times. However, this current case is quite different. R&D cuts are so deep that the US military risks paralysis in this area even if the economic climate improves. Thus, a sledgehammer versus a scalpel is being used to excise excessive spending. For example, currently there is a hold on US military attendance at scientific conferences unless a complex and expensive review is completed by multiple layers of scrutiny. While at first glance this may seem like a benign, or even wise, decision, in reality it is insignificant in the savings realized and crippling for the future. This ban stifles the exchange of ideas, destroys hard-won collaborations, halts the progression of new ideas and innovations, and abolishes the critical public-private partnerships that actually generate revenue for government R&D efforts. This single measure could cripple opportunities for a recovery even when austerity is relaxed.

The current and proposed cuts in US military R&D may make it impossible to attract young and promising investigators, thereby creating gaps in the succession of our future leaders. This impact is affecting both ends of the spectrum: with men and women who have served this country and the world finally proceeding into well-deserved retirement, the pool of talent to replace them is growing shallower and those who could normally cultivate this new talent through mentorship will be long gone. Additionally, junior military officers are now facing the conflict of furthering their scientific career versus their military one. Advancement of both aspects once logically went hand-in-hand as exceptional research and publication records were considered promotion-worthy. However, now this situation is no longer the case and promising young military scientists may be forced to abandon their research careers to diversify their military experience, thereby stunting their growth as leaders in the scientific realm. This trend is yet another strong disincentive for recruiting bright young talent to the US military. Novel medications, diagnostics, vaccines, and other life-saving measures that we have relied on from the US military take anywhere from five to 15 years to reach full licensure, and if they are not able to plant for the coming spring, in five years the pipeline can be expected to evaporate. At the current rate of increased exodus and decreased acquisition of talented professionals, the US military's contribution to global health could mostly disappear.

**Figure 1:**
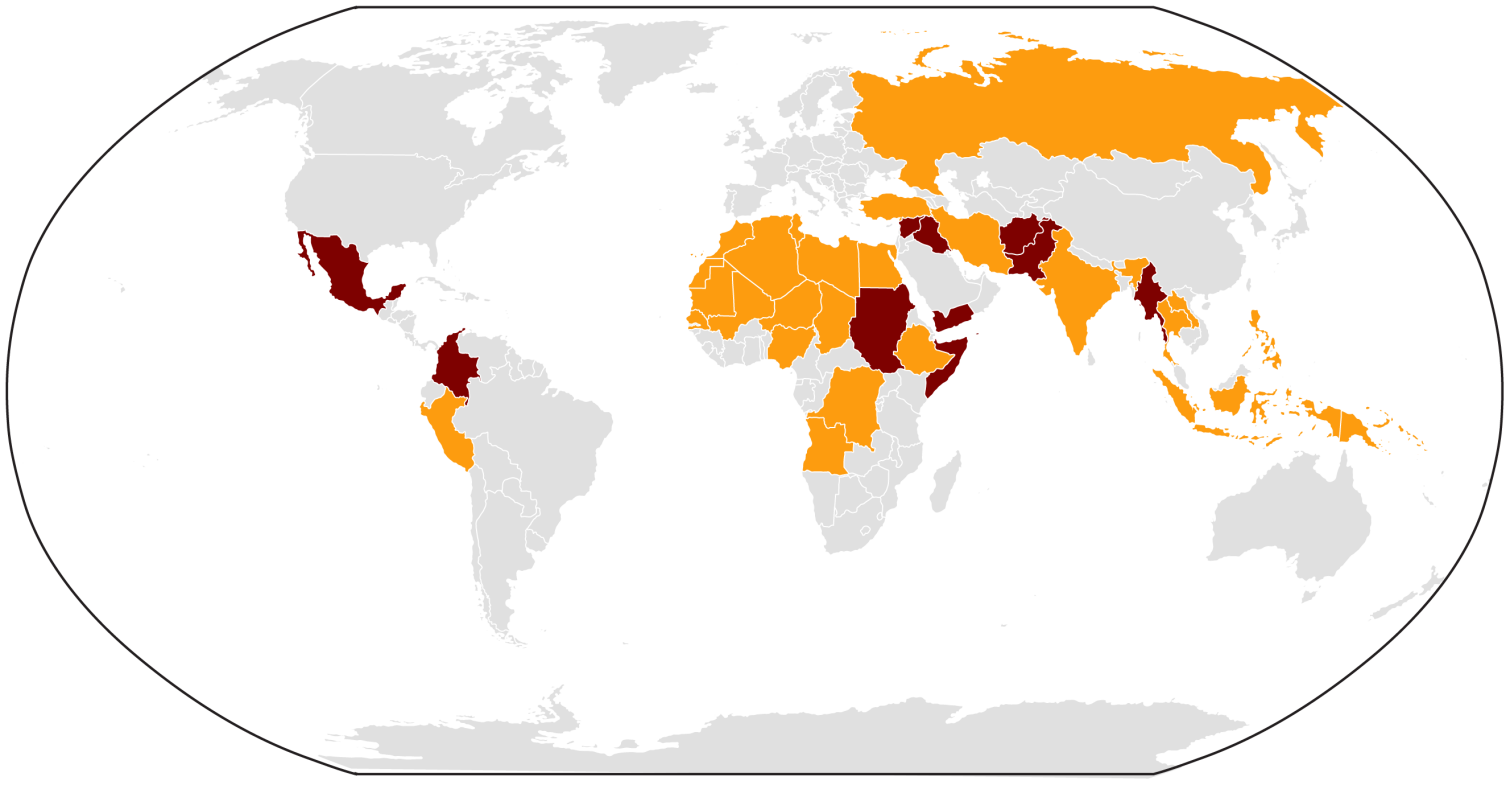
Ongoing conflicts around the world. Dark red: Major wars, 1,000+ deaths per year. Orange: Minor skirmishes and conflicts, fewer than 1,000 deaths per year.

It is almost certain that the tropical infectious disease threats we have faced in the past decade will increase should the US military enter into new theatres of operation (Figure 1). For example, an increased presence in the Pacific would bring thousands of our troops into areas endemic for dengue fever, enterovirus 71, and chikungunya, for which we currently have rudimentary preventive measures and which, based on past conflicts, could have a devastating effect on our forces. We will be facing equally grave threats if we advance further into the African continent, facing not only terrorist organizations and warring factions but old foes like malaria and other parasitic infections, viral hemorrhagic fevers, diarrhea, and respiratory diseases. Conflicts and peacekeeping in the northern reaches of Latin America would bring more malaria, CL and VL, Chagas disease, and a host of arboviral diseases. Any or all of these scenarios could play out should the US military leave the Middle East for other regions.

Though the picture that has been painted is very real and potentially bleak, it is not too late. The US Congress is urged to look to the leadership of the US military and use their knowledge and experience to reprioritize a robust WRAIR, NMRC, and the overall tropical medicine and R&D enterprise and urge a revitalized focus on increased funding and the reestablishment of rewarding, promotion-worthy career tracks in tropical medicine research.
